# Impact and Limitations of Front-Surface Reflection-Based and Anterior Segment OCT-Derived Keratometry on Refractive Prediction Accuracy in Intraocular Lens Power Calculation

**DOI:** 10.3390/jcm15155968

**Published:** 2026-07-30

**Authors:** Sumitaka Miyamoto, Kazutaka Kamiya

**Affiliations:** 1 Aira Miyamoto Eye Clinic, Aira 8995213, Japan; airamiyamotoganka@yahoo.co.jp; 2 School of Rehabilitation Sciences, Showa Medical University, Yokohama 2268555, Japan

**Keywords:** anterior segment optical coherence tomography, Fourier keratometry, keratometric index, intraocular lens power calculation, IOL constant optimization, linear interpolation

## Abstract

**Objectives:** To assess how differences in keratometric measurement principles affect intraocular lens (IOL) power calculation, we compared front-surface reflection-based (FSR) and anterior segment optical coherence tomography (AS-OCT)-derived keratometry (K) values. **Methods:** This retrospective study included 160 eyes of 160 Japanese patients who underwent cataract surgery with implantation of a CNA0T0 (Alcon) IOL between March 2023 and October 2025. FSR-based keratometry (OA-K, 2.5 mm averaged output ring) was obtained using the OA-2000 (Tomey), whereas AS-OCT-derived keratometry (CA-K, 2.5 mm single ring) and Fourier keratometry (CA-FK, 3.0 mm Fourier analysis zone) were obtained using CASIA2 (Tomey). Predicted refractions were calculated using Barrett Universal II (B-UII) and SRK/T formulas with each K value. **Results:** Mean K values were 44.14 ± 1.51 D (OA-K), 44.28 ± 1.51 D (CA-K), and 44.40 ± 1.55 D (CA-FK). With the B-UII, the mean absolute error (MAE) was lowest with OA-K (0.27 ± 0.24 D), followed by CA-K (0.28 ± 0.22 D) and CA-FK (0.31 ± 0.23 D). Corresponding MAEs with SRK/T were 0.30 ± 0.26 D, 0.31 ± 0.24 D, and 0.33 ± 0.28 D, respectively. Exploratory subgroup analyses suggested a tendency toward lower prediction accuracy in steep corneas. Differences among keratometric methods were smaller with SRK/T than with B-UII. **Conclusions:** AS-OCT-derived keratometry did not demonstrate superiority over conventional FSR-based keratometry for IOL power calculation. Further studies are warranted to determine whether AS-OCT-derived keratometry may provide clinical advantages under specific conditions.

## 1. Introduction

Optical coherence tomography (OCT)-based biometry has markedly improved postoperative refractive prediction accuracy in cataract surgery by enabling precise measurement of multiple biometric parameters, including axial length (AL), central corneal thickness (CCT), anterior chamber depth (ACD), and lens thickness (LT) [1,2]. However, keratometry (K) values are still predominantly derived using conventional front-surface reflection-based (FSR) methods. A major limitation of FSR-based keratometry is its susceptibility to tear film instability, which may compromise measurement accuracy. Both the European Society of Cataract and Refractive Surgeons (ESCRS) and the American Society of Cataract and Refractive Surgery (ASCRS) guidelines acknowledge that ocular surface disease (OSD), most commonly dry eye disease (DED), can reduce visual quality and adversely affect refractive measurements, and recommend appropriate management of the ocular surface prior to cataract surgery [3,4].

For anterior segment OCT (AS-OCT), the first commercially available device designed for anterior segment imaging was the Visante OCT (Carl Zeiss Meditec, Jena, Germany), introduced around 2005 [5]. Fourier-domain anterior segment OCT was subsequently introduced with the CASIA system (Tomey Corporation, Nagoya, Japan) in 2008, representing the first Fourier-domain OCT specifically developed for anterior segment applications. The technology has since evolved, and the current CASIA2 system (Tomey) provides higher scanning speed and improved axial resolution, enabling detailed characterization of the anterior corneal surface and Fourier-based keratometry [6,7]. Unlike conventional FSR-based keratometry, AS-OCT reconstructs the anterior corneal surface from cross-sectional OCT images and may therefore be less affected by tear film instability.

In conventional keratometry, corneal power is not measured directly but is calculated from the anterior corneal curvature using a standardized keratometric refractive index that approximates the contribution of the posterior corneal surface. Therefore, differences in keratometric derivation principles may influence not only measurement repeatability but also the refractive utility of K values for IOL power calculation.

Therefore, this study compared FSR-based and AS-OCT-derived keratometry measurements to evaluate their impact on IOL power prediction, using the Barrett Universal II (B-UII) and SRK/T formulas, which are among the most widely used IOL power calculation formulas in Japan [8]. To our knowledge, few studies have evaluated the accuracy of IOL power calculation using K values derived from AS-OCT measurements in accordance with the protocols proposed by Hoffer et al. [9,10]. Previous studies using CASIA2 have mainly focused on agreement and repeatability of corneal parameter measurements between devices [11,12,13]. Accordingly, the present study aimed to clarify how differences in keratometric measurement principles influence refractive prediction accuracy in contemporary IOL power calculation.

## 2. Materials and Methods

### 2.1. Study Design

This retrospective observational study was conducted at a single institution and included patients who underwent cataract surgery with implantation of a Clareon monofocal IOL (CNA0T0, Alcon Laboratories, Geneva, Switzerland; manufacturer-recommended A constant: 119.1) between March 2023 and October 2025. Ethical approval was obtained from the Kyoto Expert Ethics Committee, which is registered with the Ministry of Health, Labor and Welfare of Japan (approval ID: KEEC-21035; approved 11 November 2025). All data were anonymized prior to analysis, and patients were provided with an opt-out opportunity.

### 2.2. Setting

The biometric parameters evaluated were AL, CCT, horizontal corneal diameter (HCD, white-to-white, WTW), ACD, LT, and K values. FSR-based keratometry (OA-K, 2.5 mm averaged output ring) and AL were obtained using the OA-2000 (Tomey), whereas AS-OCT-derived keratometry (CA-K, 2.5 mm single ring), Fourier keratometry (CA-FK, 3.0 mm Fourier analysis zone), and all biometric parameters other than AL were obtained using CASIA2 (Tomey). Hereafter, OA-K, CA-K, and CA-FK are collectively referred to as “keratometric measurement methods (KMs)”. All eyes were measured using both devices on the same day. Predicted refractions were calculated using the B-UII formula and the SRK/T formula implemented in the built-in software of CASIA2 (software version 50.7c.01). Postoperative examinations were performed 3 months after surgery. Corrected visual acuity (CVA) and subjective refraction, including spherical and cylindrical components, were assessed by an orthoptist at a testing distance of 5 m, and spherical equivalent refraction was subsequently calculated. Prediction error (PE) was defined as the difference between the predicted refraction for each KM and the postoperative spherical equivalent refraction. Thus, all biometric parameters were held constant while only the KM was varied. This study design enabled isolation of the effect of KMs on refractive prediction accuracy by minimizing the influence of other biometric variables.

### 2.3. IOL Power Calculation Formula Protocol

The Barrett Universal II formula is a modern theoretical IOL power calculation formula based on paraxial Gaussian optics. A key feature of this formula is that it models the effective lens position as a function of multiple biometric parameters and explicitly accounts for changes in principal plane location with different IOL powers. In addition, it incorporates a lens geometry model that transitions from biconvex to meniscus configurations, allowing stable refractive prediction across a wide range of axial lengths without requiring separate high-myopia correction factors [14].

In contrast, the SRK/T formula is a third-generation regression-based formula that estimates effective lens position primarily from axial length and keratometry, with internal empirical corrections for retinal thickness and anterior chamber depth prediction [15].

Because keratometry contributes differently to effective lens position estimation in these two formulas, variations in KM may differentially affect refractive prediction accuracy.

### 2.4. Device Protocol

OA-2000 (Tomey, version 5B1/7P) is based on Fourier-domain technology and utilizes swept-source OCT with a variable-wavelength laser at 1060 nm to perform 41 horizontal A- and V-scans. K values were measured using the Placido disk-based topography technique by projecting 9 rings, each with 256 points, onto a 5.5 mm zone of the cornea, obtaining diameters of 2.5 mm with a refractive index of 1.3375 [2,16,17].

Meanwhile, CASIA2 (Tomey, version 50.7c.01) is the second generation of swept-source AS-OCT with a wavelength of 1310 nm and an acquisition speed of 50,000 A-scans/s. In the standard keratometry mode, CASIA2 measures the anterior corneal surface using a single ring with 32 sampling points at diameters of 2.5 mm or 3.0 mm, using the same standardized keratometric refractive index of 1.3375. In contrast, the Fourier keratometry (CA-FK) mode analyzes the anterior corneal surface within a 3.0 mm zone using 75 concentric rings comprising a total of 2400 sampling points, enabling high-density corneal power assessment [7,11,12,13].

**Figure 1:**
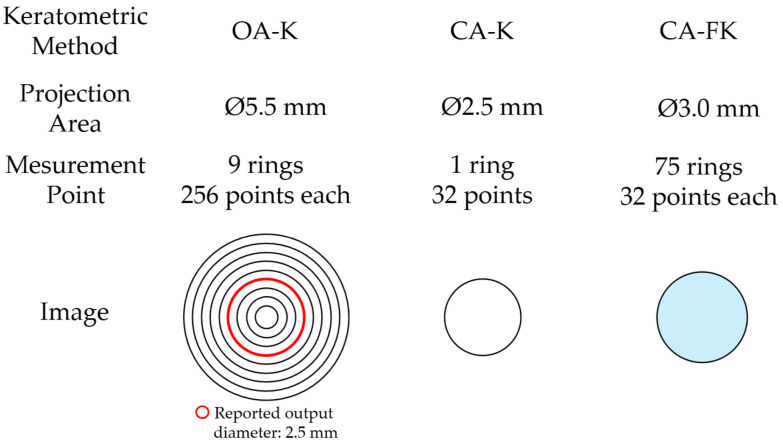
Schematic comparison of keratometric methods used in this study. OA-K, CA-K, and Fourier keratometry (CA-FK) differ in measurement area and sampling density. OA-K calculates keratometry using nine concentric rings within a 5.5 mm area, CA-K uses a single ring with a diameter of 2.5 mm, and CA-FK calculates keratometry by averaging 75 rings, each composed of 32 points, within a 3.0 mm area.

The differences in KM principles, including measurement area and sampling density, are summarized schematically in Figure 1. Because the OA-2000 and CASIA2 systems are interoperable, manual data input is unnecessary, facilitating reproducible implementation of the present study protocol.

### 2.5. Optimization of IOL Constants Using Linear Interpolation

**Figure 2:**
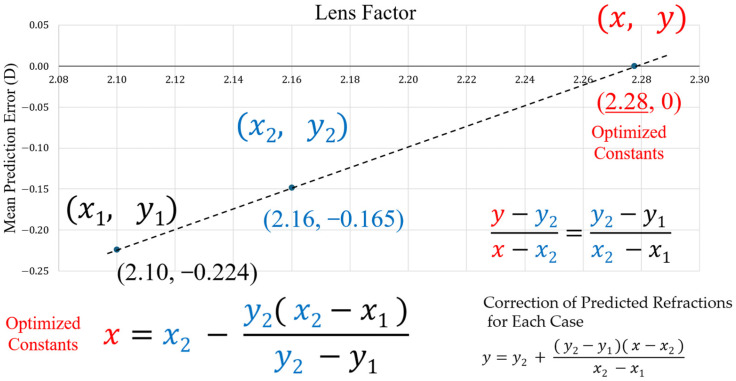
Conceptual diagram of linear interpolation applied for optimization of IOL constants. Two known IOL constants and their corresponding mean prediction errors are plotted, and linear interpolation is performed between the two points. The optimized A-constant is determined as the x-coordinate at which MPE = 0.

In this study, IOL constants were optimized using the IOL Optimization Planner (Japanese Patent Application No. 2025-99851), a calculation tool developed using Microsoft Excel based on the previously reported linear interpolation (LI) method [17]. Briefly, as illustrated in Figure 2, two preset constants and their corresponding mean prediction errors were used to interpolate the constant at which the mean prediction error became 0. In this study, Lens Factor (LF) values of 2.10 and 2.16 were used for the B-UII, and A constants of 119.43 and 119.54 were used for the SRK/T. Optimized IOL constants were calculated separately for each KM. This approach enabled accuracy comparisons in accordance with the standardized protocol for IOL power calculation proposed by Hoffer et al. [9,10].

### 2.6. Inclusion and Exclusion Criteria

The inclusion criteria were: cataract surgery performed by a single surgeon; an upper transconjunctival single-plane incision at 90°; complete intracapsular fixation of the IOL; and absence of intraoperative or postoperative complications. Only eyes with corrected visual acuity (CVA) of 20/40 or better were eligible for inclusion, and one eye per patient was analyzed. When both eyes satisfied the inclusion criteria, the eye with better CVA was selected. Exclusion criteria included a history of refractive surgery or corneal disease, such as pterygium or corneal endothelial disorders.

### 2.7. Primary Outcome Measures

We calculated the mean K value for each KM. For each eye, the absolute prediction error (APE) was calculated for each formula and KM. From these values, we calculated the mean absolute prediction error (MAE), median absolute prediction error (MedAE), standard error, the proportions of eyes with an APE within 0.25 D and 0.50 D (abbreviated as “the rate <0.25 D, <0.5 D”), and the 95% confidence interval (CI) for the MAE.

### 2.8. Statistical Analysis

Sample size estimation for comparisons using the Friedman test was performed with G*Power version 3.1.9.7 using an initial subset of 30 eyes collected during the early phase of the study. Assuming an effect size of f = 0.25, a significance level of α = 0.05, and a statistical power of 0.80, the estimated required sample size ranged from 11 to 27 eyes depending on the assumed correlation among repeated measures. Statistical analyses were conducted using JAMOVI version 2.3.28.0 (The Jamovi Project) and R version 4.4.2. Data management and preliminary calculations were performed using Microsoft Excel (version 2512, Build 16.0.19530.20144, 64-bit; Microsoft 365).

The normality of APE distributions was assessed using the Shapiro–Wilk test for each combination of formula and KM. For normally distributed data, repeated-measures ANOVA was applied to compare MAE among KMs, whereas the Friedman test was used for non-normally distributed data. Comparisons of the rates <0.25 D and <0.50 D among KMs were performed using Cochran’s Q test, while comparisons between formulas were conducted using McNemar’s test. When significant differences among KMs were identified, post hoc pairwise analyses were performed using the Durbin–Conover procedure or McNemar’s test with Bonferroni correction.

## 3. Results

### 3.1. Participant Characteristics and Measurements

**Figure 3:**
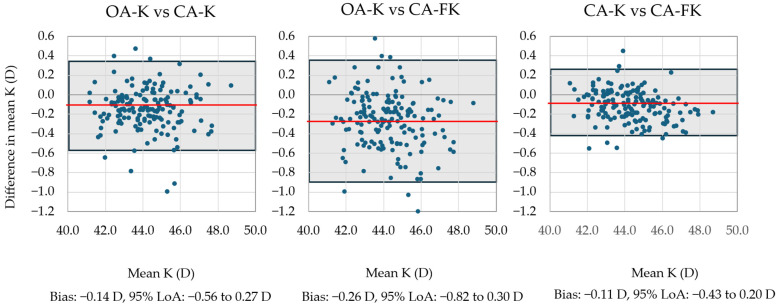
Bland–Altman plots comparing keratometric methods. The red line indicates the mean difference (bias), and the gray shaded areas indicate the 95% limits of agreement. Pairwise comparisons were performed among OA-K, CA-K, and CA-FK.

This study included 160 eyes of 160 Japanese patients (mean age ± standard deviation [SD]: 72.5 ± 9.2 years; male, 50.0%). The mean postoperative follow-up duration was 95.6 ± 9.5 days (range: 83–132 days). The common biometric parameters and K values for each KM are summarized in Table 1. All K values showed normal distributions (Shapiro–Wilk test, *p* > 0.05); therefore repeated-measures ANOVA was applied. Significant differences were observed among all KM combinations (*p* < 0.001). Bland–Altman plots comparing KMs are shown in Figure 3. Systematic inter-method differences (bias) and the corresponding 95% limits of agreement were observed for all KM combinations. The agreement between CA-K and CA-FK was relatively higher, with a smaller bias and narrower limits of agreement, whereas comparisons involving OA-K showed wider variability.

### 3.2. Comparative Analysis of Absolute Prediction Errors for Each Formula and Keratometric Method

#### 3.2.1. For All Cases

**Figure 4:**
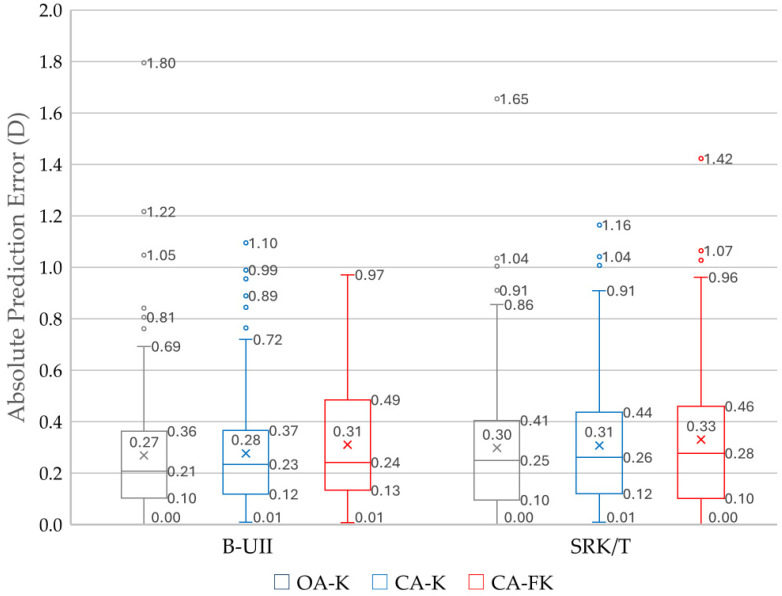
Box-and-whisker plots of absolute prediction error (APE) for each keratometric method (OA-K, CA-K, and CA-FK) in the Barrett Universal II and SRK/T formulas. Boxes indicate the interquartile range with the median shown as a horizontal line; whiskers represent the range excluding outliers.

Table 2 summarizes prediction accuracy metrics for each formula and KM across all cases. P-KM and P-formula represent *p*-values for comparisons among KMs and between formulas, respectively. Overall, prediction accuracy tended to be more favorable with B-UII than with SRK/T. In both formulas, OA-K tended to show more favorable prediction accuracy than CA-K and CA-FK, but inter-method differences were more pronounced with B-UII, especially in MAE and in the rates <0.25 D and <0.5 D. Figure 4 presents box-and-whisker plots of APE for each formula and KM. Differences among KMs tended to be larger with B-UII, whereas changes in APE distributions across KMs were relatively smaller with SRK/T.

#### 3.2.2. Subgroup Analysis

Because standardized cutoff values for classifying flat and steep corneas have not yet been established, an exploratory subgroup analysis was performed. Using OA-K as the reference K value, subgroup selection was guided by the distribution of OA-K values together with the results of the sample size analysis. Based on these considerations, eyes representing the lower and upper extremes of the OA-K distribution were included, resulting in two subgroups of 25 eyes each (Flat: 41.14–42.62 D; Steep: 45.55–48.74 D).

Table 3 summarizes subgroup analyses according to corneal curvature. Compared with the overall cohort, refractive prediction accuracy tended to be more favorable in the Flat group and less favorable in the Steep group across both formulas. This tendency was not fully eliminated even with B-UII.

Comparisons among KMs showed a tendency similar to that observed in the overall analysis, with relatively lower prediction accuracy for CA-FK. However, differences in prediction accuracy between OA-K and CA-FK were smaller with SRK/T than with B-UII.

## 4. Discussion

At the time of study design, we hypothesized that AS-OCT-derived keratometry would improve refractive prediction accuracy because it would be less affected by ocular surface instability than FSR-based keratometry. However, contrary to our hypothesis, FSR-based keratometry showed slightly lower MAE than AS-OCT-derived keratometry. Although the difference was only approximately 0.04 D, the results indicate that replacing FSR-based keratometry with AS-OCT-derived K values did not improve refractive prediction accuracy, and the clinical difference among KMs was minimal.

One possible explanation relates to differences in KM principles (Figure 1). OA-K is based on FSR with spatial averaging, CA-K relies on single-ring measurement, and CA-FK analyzes zonal corneal power using Fourier-based decomposition rather than simple averaging. CA-FK incorporates a larger number of measurement points and provides a more detailed representation of corneal shape; however, such detailed shape information may not necessarily translate into the most appropriate K value for IOL power calculation.

As illustrated in Figure 4, the differences in refractive prediction accuracy between OA-K and CA-FK appeared smaller with SRK/T than with B-UII. These findings suggest that optimization of IOL constants is relatively effective at compensating for differences among KMs in simpler formulas such as SRK/T, which depend primarily on AL and K values. B-UII incorporates additional parameters, including ACD, LT, and CCT, in addition to AL and K values. Therefore, dependence on K values in refractive prediction may be relatively reduced compared with simpler formulas such as SRK/T. This may partially compensate for differences in K values among KMs, thereby improving overall refractive prediction accuracy. On the other hand, we believe that optimization based solely on adjustment of a single IOL constant may be insufficient for contemporary advanced IOL calculation formulas [18].

The exploratory subgroup analysis demonstrated that refractive prediction accuracy tended to deteriorate in eyes with steeper corneas, suggesting that corneal shape itself may influence the appropriateness of K values used for IOL power calculation. K values are calculated from the anterior corneal radius of curvature using a standardized keratometric refractive index (1.3375 in the devices used in the present study) [19,20]. This assumption is known to become less accurate in post–keratorefractive surgery eyes, such as those undergoing LASIK, where the anterior–posterior corneal relationship is altered [21,22]. Therefore, a similar limitation of this assumption may also exist in eyes with steeper corneas.

Based on the lack of consistent differences among KMs and the reduced accuracy observed in steep corneas, we believe that these findings arose from the use of a uniform keratometric refractive index across different corneal shapes and KMs rather than from the measurement devices and methods themselves. Optimization of IOL constants tailored to each device and formula remains important [17,23]. However, given the limitations of single IOL constant optimization, we hypothesize that refinement of K value representation may further improve refractive prediction accuracy in future studies.

The present study has several limitations. First, this was a retrospective single-center study conducted in a homogeneous cohort of Japanese patients who underwent cataract surgery performed by a single surgeon using a single monofocal IOL model. Although this design reduced potential confounding factors, it may limit the generalizability of the findings to other ethnic populations, surgeons, surgical settings, and IOL models. Second, the subgroup analyses were exploratory and based on the upper and lower 25 eyes according to K values rather than clinically established thresholds; therefore, these findings should be interpreted with caution and require confirmation in larger prospective studies using standardized cutoff values when available. Third, although keratometry derived from AS-OCT is theoretically less affected by OSD than that derived from FSR, ocular surface conditions were not objectively evaluated in this study. Future studies are needed to determine the influence of ocular surface conditions on keratometric measurements and to further clarify the potential clinical utility of AS-OCT-derived keratometry.

Nevertheless, because all biometric parameters other than keratometric measurements were intentionally held constant, we believe that the present study design should be readily applicable to future validation studies involving multiple surgeons, institutions, IOL models, measurement devices, and more diverse patient populations.

## 5. Conclusions

Replacing FSR-based keratometry with AS-OCT-derived K values did not improve refractive prediction accuracy, and the clinical difference among KMs was minimal. These findings suggest that detailed AS-OCT-based corneal shape analysis may not necessarily translate into the most appropriate K value for IOL power calculation.

## 6. Patents

The IOL Optimization Planner introduced in this study has been officially filed for patent protection in Japan as of 16 June 2025 (Japanese Patent Application No. 2025-99851).

## Figures and Tables

**Table 1:** Summary of patient characteristics, biometric parameters, and keratometry values for each keratometric method.

**Age**		72.5 ± 9.2
**Sex (eyes)**		Men 80, Women 80
**Follow-up duration**		95.6 ± 9.5 days
**Common Biometric Parameters**		
**OA-2000**	**AL**	24.15 ± 1.56 mm
**CASIA2**	**CCT**	522 ± 35 µm
	**ACD**	3.18 ± 0.38 mm
	**LT**	4.65 ± 0.46 mm
	**HCD**	11.56 ± 0.43 mm
**Keratometric Method**		
**OA-2000**	**OA-K**	44.14 ± 1.51 D
**CASIA2**	**CA-K**	44.28 ± 1.51 D
	**CA-** **FK**	44.40 ± 1.55 D
***p*** **-value**		<0.001
**Post hoc**		<0.001 for all combinations

Normality was assessed using the Shapiro–Wilk test. Depending on the results, repeated-measures ANOVA was used for group comparisons among keratometric methods. AL: Axial length; ACD: anterior chamber depth; LT: lens thickness; CCT: central corneal thickness; HCD: horizontal corneal diameter; OA-K: keratometry obtained using the OA-2000; CA-K: keratometry obtained using CASIA2 in standard keratometry mode; CA-FK: keratometry obtained using CASIA2 in Fourier keratometry mode.

**Table 2:** Summary of prediction accuracy metrics for each formula and keratometric method across all cases.

All Cases (*n* = 160)		OA-K	CA-K	CA-FK	*P*-KM
**IOL constant**	**Barrett**	2.18	2.28	2.35	
**(LF, A constant)**	**SRK/T**	119.39	119.54	119.62	
**MAE ± SD**	**Barrett**	0.27 ± 0.24	0.28 ± 0.22	0.31 ± 0.23	<0.01 (1)
**(D)**	**SRK/T**	0.30 ± 0.26	0.31 ± 0.24	0.33 ± 0.28	<0.01 (2)
	***P*** **-formula**	<0.05	<0.05	0.278	
**The rate <0.25 D**	**Barrett**	55.6	51.9	50.0	0.362
**(%)**	**SRK/T**	49.4	48.1	47.5	0.856
	***P*** **-formula**	0.059	0.289	0.505	
**The rate <0.5 D**	**Barrett**	87.5	83.8	76.9	<0.001 (3)
**(%)**	**SRK/T**	82.5	81.3	78.8	0.264
	***P*** **-formula**	0.071	0.371	0.549	
**Standard Error**	**Barrett**	0.019	0.017	0.018	
	**SRK/T**	0.021	0.019	0.022	
**MedAE**	**Barrett**	0.21	0.24	0.25	
	**SRK/T**	0.25	0.27	0.28	
**95% CI**	**Barrett**	0.23–0.31	0.25–0.31	0.28–0.35	
**(MAE, %)**	**SRK/T**	0.26–0.34	0.27–0.35	0.29–0.37	
**Post hoc**	(1) OA-K vs. CA-FK (<0.01), (2) OA-K vs. CA-K, CA-FK (<0.05)				
**comparisons:**	(3) OA-K vs. CA-FK (<0.05)				

Statistical analyses were performed using the Friedman test or Cochran’s Q test for comparisons among keratometric methods, and the Wilcoxon signed-rank test or McNemar’s test for comparisons between formulas. Post hoc analyses were conducted with Bonferroni correction when appropriate. OA-K: Keratometry obtained using OA-2000; CA-K: keratometry obtained using CASIA2 in standard keratometry mode; CA-FK: keratometry obtained using CASIA2 in Fourier keratometry mode; *P*-KM: *p*-values for comparisons among keratometric methods; *P*-formula: *p*-values for comparisons between formulas; Barrett: Barrett Universal II formula; MAE: mean absolute prediction error; The rate <0.25 D, <0.5 D: the rates of eyes with absolute prediction error within 0.25 D and 0.5 D; MedAE: median absolute prediction error; CI: confidence interval.

**Table 3:** Summary of prediction accuracy metrics for each formula and keratometric method stratified by corneal curvature.

Comparison by Formula					
Flat Cornea (*n* = 25)		OA-K	CA-K	CA-FK	*P*-KM
**MAE ± SD**	**Barrett**	0.20 ± 0.14	0.20 ± 0.13	0.25 ± 0.19	0.205
**(D)**	**SRK/T**	0.29 ± 0.16	0.29 ± 0.19	0.31 ± 0.25	0.527
	***P*** **-formula**	<0.001	<0.05	0.08	
**The rate <0.5 D**	**Barrett**	92.0	96.0	88.0	0.368
**(%)**	**SRK/T**	88.0	84.0	84.0	0.819
	***P*** **-formula**	0.317	0.083	0.564	
**MedAE**	**Barrett**	0.16	0.20	0.21	
	**SRK/T**	0.29	0.24	0.28	
**Steep Cornea (*n* = 25)**		**OA-K**	**CA-K**	**CA-FK**	**P-KM**
**MAE ± SD**	**Barrett**	0.30 ± 0.25	0.36 ± 0.28	0.37 ± 0.24	0.237
**(D)**	**SRK/T**	0.38 ± 0.28	0.43 ± 0.31	0.39 ± 0.30	<0.05 (1)
	***P*** **-formula**	<0.05	0.085	0.518	
**The rate <0.5 D**	**Barrett**	76.0	76.0	64.0	0.165
**(%)**	**SRK/T**	68.0	68.0	68.0	NaN
	***P*** **-formula**	0.317	0.317	0.564	
**MedAE**	**Barrett**	0.19	0.27	0.33	
	**SRK/T**	0.36	0.30	0.30	
**Comparison by Corneal Curvature** (Eyes: Flat cornea 25, Steep cornea 25)					
**Barrett Universal II**		**OA-K**	**CA-K**	**CA-FK**	***P*** **-KM**
**MAE ± SD**	**Flat**	0.20 ± 0.14	0.20 ± 0.13	0.25 ± 0.19	0.205
**(D)**	**Steep**	0.30 ± 0.25	0.36 ± 0.28	0.37 ± 0.24	0.237
	***P*** **-curvature**	0.118	<0.05	0.055	
**The rate <0.5 D**	**Flat**	92.0	96.0	88.0	0.368
**(%)**	**Steep**	76.0	76.0	64.0	0.165
	***P*** **-curvature**	0.247	0.098	0.095	
**MedAE**	**Flat**	0.16	0.20	0.21	
	**Steep**	0.19	0.27	0.33	
**SRK/T**		**OA-K**	**CA-K**	**CA-FK**	**P-KM**
**MAE ± SD**	**Flat**	0.29 ± 0.16	0.29 ± 0.19	0.31 ± 0.25	0.527
**(D)**	**Steep**	0.38 ± 0.28	0.43 ± 0.31	0.39 ± 0.30	<0.05 (1)
	***P*** **-curvature**	0.304	0.111	0.473	
**The rate <0.5 D**	**Flat**	88.0	84.0	84.0	0.819
**(%)**	**Steep**	68.0	68.0	68.0	NaN
	***P*** **-curvature**	0.171	0.321	0.321	
**MedAE**	**Flat**	0.29	0.24	0.28	
	**Steep**	0.36	0.30	0.30	
**Post hoc**	(1) CA-K vs. CA-FK (<0.05)				
**comparisons:**					

Statistical analyses were performed using the Friedman test or Cochran’s Q test for comparisons among keratometric methods, the Wilcoxon signed-rank test or McNemar’s test for comparisons between formulas and the Mann–Whitney U test or Fisher’s exact test for comparisons between corneal curvature. Post hoc analyses were conducted with Bonferroni correction when appropriate. OA-K: Keratometry obtained using OA-2000; CA-K: keratometry obtained using CASIA2 in standard keratometry mode; CA-FK: keratometry obtained using CASIA2 in Fourier keratometry mode; *P*-KM: *p*-values for comparisons among keratometric methods; *P*-formula: *p*-values for comparisons between formulas; *P*-curvature: *p*-values for comparisons between Flat and Steep groups; NaN: not applicable due to identical values in all groups; MAE: mean absolute prediction error; The rate <0.5 D: the rates of eyes with absolute prediction error within 0.5 D; MedAE: median absolute prediction error.

## Data Availability

The data supporting the findings of this study are available from the corresponding author upon reasonable request. Data are not publicly available due to privacy restrictions.
